# Survival outcome of local versus radical resection for jejunoileal gastrointestinal stromal tumors: a propensity score-matched population-based analysis

**DOI:** 10.1007/s00384-023-04548-w

**Published:** 2023-10-19

**Authors:** Shangcheng Yan, Wei Peng, Ming Cheng, Jingyu Zhang, Tianhua Liu, Mengchao Sheng, Rui Ren, Qiang Chen, Wei Gong, Yongyou Wu

**Affiliations:** 1 Department of Gastrointestinal Surgery, Second Affiliated Hospital of Soochow University, Suzhou, China; 2 Department of Gastroenterology and Minimally Invasive Surgery, Juntendo University Hospital, Tokyo, 113-8431 Japan

**Keywords:** Gastrointestinal stromal tumors, Local resection, Small intestine, Jejunoileal, Survival

## Abstract

**Purpose:**

Survival after local resection (LR) versus radical resection (RR) has been revealed comparable for patients with rectal and duodenal gastrointestinal stromal tumors (GISTs), but is unknown for jejunoileal (JI) GISTs. This study aimed to compare the long-term survival between patients with JI GISTs who underwent LR and RR, and to find out the prognostic factors for JI GISTs.

**Methods:**

Patients diagnosed with JI GISTs in 1975–2019 were identified from Surveillance, Epidemiology, and End Results (SEER) database and grouped according to surgical modality. Propensity score matching (PSM) was performed to balance the LR and RR groups. Overall survival (OS) and disease-specific survival (DSS) were compared in the full and matched cohorts using Kaplan–Meier (KM) analysis. Subgroup sensitivity analyses were also performed. Risk factors associated with DSS were analyzed in multivariate Cox analysis following model selection.

**Results:**

1107 patients diagnosed with JI GISTs were included in the study cohort. After PSM, OS and DSS were comparable in LR and RR groups. Consistently, the two groups had similar DSS in all subgroup analyses. Moreover, multivariate Cox analysis identified lymphadenectomy, older age, larger tumor size, distant metastasis, high and unknown mitotic rate, but not LR, as independent prognostic risk factors for JI GISTs.

**Conclusions:**

We conducted the first population-based comparison between the effect of different surgical modes on survival for patients with JI GISTs. LR can be carried out safely without compromising oncological outcome, and should be considered as a treatment option in selected patients with JI GISTs.

**Supplementary Information:**

The online version contains supplementary material available at 10.1007/s00384-023-04548-w.

## Introduction

Though gastrointestinal stromal tumors (GISTs) are rare malignant tumors, they represent the most common sarcomas of the digestive system. GISTs could occur throughout the gastrointestinal tract and extragastrointestinally, but were found most frequently in stomach (50–60%), followed by jejunum and ileum (20–30%) [1, 2]. The most important prognostic factors for GISTs are tumor size, mitotic rate and tumor rupture [3, 4], but tumor site is also related with the risk of recurrence and prognosis since the nature of GISTs differ among locations [5]. Although jejunoileal (JI) GISTs are generally considered more aggressive than gastric GISTs, their characteristics remain controversial [6–8]. Indeed, only a few studies focused exclusively on JI GISTs with small sample sizes [8–10].

Surgery remains the only curative modality of therapy for GISTs if negative margins are ensured and tumor rupture are avoided [4]. Expanding resection margin and lymphadenectomy is unnecessary for GISTs since they rarely invade the adjacent organs or regional lymph nodes [11]. Moreover, studies have found that the positive microscopic margin (R1) may not influence survival for GISTs significantly, with or without adjuvant targeted therapy [12, 13]. Therefore, local resection (LR) such as wedge or segmental resection might be sufficient for GISTs whereas extended radical resection (RR) should be avoided [4, 14]. Comparable survival outcomes of LR versus RR have been found in duodenal and rectal GISTs [15–17], but it is questionable whether these findings could be expanded to other sites such as jejunum and ileum.

The aim of this study was to compare the survival outcome between patients with JI GISTs who underwent LR and RR. We also sought to identify the prognostic factors for JI GISTs.

## Methods

### Patient selection

Given the relatively low incidence of JI GISTs, we used Surveillance, Epidemiology, and End Results (SEER) database which collects data from population-based cancer registries that cover 34% of the U.S. population. This study used publicly available de-identified data involving no human participants, and thus was granted exemption by the institutional review board at Second Affiliated Hospital of Soochow University.

**Fig. 1:**
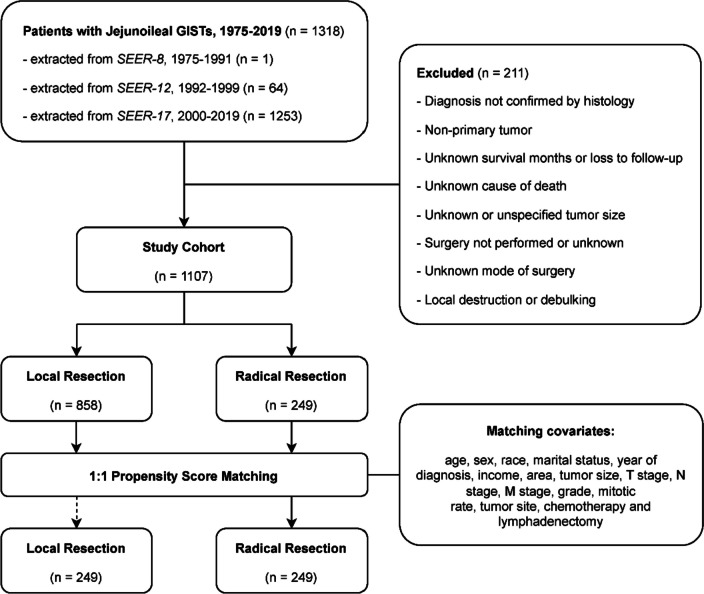
Patient selection process. GISTs: gastrointestinal stromal tumors; SEER: Surveillance, Epidemiology, and End Results

Patient selection is outlined in Fig. 1. Patients diagnosed with JI (International Classification of Diseases for Oncology, 3rd Edition [ICD-O-3] topography codes C17.1 and C17.2) GISTs (ICD-O-3 histology code 8936/3) from January 1 1975 through December 31 2019 were identified from the SEER database using SEER*Stat software version 8.4.0.1. Database names and detailed selection statements used for this study is provided in Supplementary Table 1. Patients were excluded for: (1) diagnosis not confirmed by histology, (2) non-primary tumor, (3) unknown survival months or loss to follow-up, (4) unknown cause of death, (5) unknown or unspecified tumor size, (6) surgery not performed or unknown, (7) unknown mode of surgery, or (8) local tumor destruction or debulking.

### Study variables

Predictor variables (age, sex, race, marital status, year of diagnosis, income, area, tumor size, T stage, N stage, M stage, grade, mitotic rate, tumor site, chemotherapy, lymphadenectomy and mode of surgery), survival time and outcome variables (all-cause death [ACD] and disease-specific death [DSD]) used in this study were collected from SEER database and recoded.

T, N, and M stages were redefined according to the newest 8^th^ edition of American Joint Committee on Cancer (AJCC) staging manual [3]. N0 was defined as no or unknown regional lymph node metastasis (LNM), and M0 was defined as no or unknown distant metastasis. Mitotic rate was recorded in SEER database since 2004, and was defined as low (≤ 5 mitoses per 5 mm^2^ or per 50 high-power field [HPF]) or high (> 5 mitoses per 5 mm^2^ or per 50 HPF) for patients with these data available, otherwise as unknown. Moreover, we grouped age (< 45, 45–79 and ≥ 80 years), tumor size (< 89 and ≥ 89 mm), T stage (T1-2, T3 and T4) and grade (I, II-IV and unknown) for survival analysis. Lymphadenectomy was defined by scope of regional lymph node surgery as no/unknown versus yes.

Mode of surgery is defined by site-specific surgery codes in SEER database. LR was defined as local tumor excision, or simple/partial removal of primary site (code 35–50 before 1998; code 20–30 since 1998). RR was defined as total removal of primary site, or partial/total removal of primary site with partial/total removal of other organs (code 60 before 1998; code 40 and 60 since 1998) [18]. Survival time was defined as months from diagnosis to ACD or last follow-up (December 31 2019). All patients in the study cohort completed full follow-up. DSD was identified by “Dead (attributable to this cancer dx)” in “SEER cause-specific death classification”.

### Statistical analysis

Patients were divided into LR and RR groups based on their surgical modality. A 1:1 ratio propensity score matching (PSM) was performed based on age, sex, race, marital status, year of diagnosis, income, area, tumor size, T stage, N stage, M stage, grade, mitotic rate, tumor site, chemotherapy and lymphadenectomy to the two groups using “optimal” method in R package “MatchIt”. Before and after PSM, continuous variables were compared with unpaired Student’s *t* tests, while categorical variables were compared with *Chi*-square tests.

Survival analyses were performed using R package “survival”. Kaplan–Meier (KM) survival analysis with the log-rank test was used to assess the differences in overall survival (OS) and disease-specific survival (DSS) between the LR and RR groups. Survival probabilities at 5 year and 10 year were compared using “fixtdiff” function of R package “bpcp”. Hazard ratio (HR) for DSS with 95% confidence interval (CI) between the two groups was computed in the matched cohort and subgroups using the log-rank test.

Then, HR for DSS with 95% CI was estimated for each predictor variable using univariate Cox proportional hazards regression model (with Breslow’s ties). Furthermore, multivariate Cox models were fitted for mode of surgery, together with all possible combinations of variables with univariate *p* value < 0.1. To prevent overfitting, we performed automated model selection based on corrected Akaike Information Criterion (AICc) using “dredge” function in R package “MuMIn”. Models with ΔAICc (difference in AICc between a model and the model with the lowest AICc) < 2 were considered informationally equivalent. Predictor variables included in the simplest model (having the least number of variables) with the relatively smallest AICc were fitted into multivariate analysis, along with mode of surgery.

Statistical analysis was performed with R (version 4.2.1, R Core Team, Vienna, Austria). Best cutoff values for continuous variables were determined with X-tile (version 3.6.1, Yale University School of Medicine, New Haven, USA) (Supplementary Fig. 1). A two-sided *p* value < 0.05 was considered statistically significant. Values were presented as mean ± standard deviation for continuous variables and as number (%) for categorical variables.

## Results

### Patient characteristics and propensity score matching

Originally, 1318 patients with JI GISTs were extracted from SEER database, accounting for 35.6% of the total cases with small intestinal GISTs. Through patient selection, 1107 patients diagnosed from 1990 through 2019 were included in the final cohort (Fig. 1). The mean age at diagnosis for the full study cohort was 61.1 ± 14.3 years (Table 1). LR was conducted in 858 (77.5%) patients. The proportion of patients underwent LR increased from 70.8% (1990–2003) to 76.8% (2004–2011) and 80.5% (2012–2019).

**Table 1:** Comparison of clinicopathological characteristics between local resection and radical resection groups before and after propensity score matching

**Variable**	**Before PSM**			**After PSM**	
	**LR (n = 858)**	***p* value**	**RR (n = 249)**	***p* value**	**LR (n = 249)**
Age (years), mean ± SD	60.79 ± 14.25	0.202	62.11 ± 14.58	0.271	63.53 (14.12)
Sex, n (%)		0.126		1.000	
Male	497 (57.9)		130 (52.2)		130 (52.2)
Female	361 (42.1)		119 (47.8)		119 (47.8)
Race, n (%)		0.354		0.660	
White	652 (76.0)		198 (79.5)		193 (77.5)
Black	71 (8.3)		21 (8.4)		27 (10.8)
Other/UNK	135 (15.7)		30 (12.0)		29 (11.6)
Marital status, n (%)		0.037		0.991	
Married	540 (62.9)		146 (58.6)		141 (56.6)
Single	128 (14.9)		50 (20.1)		54 (21.7)
Divorced	66 (7.7)		13 (5.2)		14 (5.6)
Widowed	81 (9.4)		33 (13.3)		33 (13.3)
Other/UNK	43 (5.0)		7 (2.8)		7 (2.8)
Year of diagnosis, n (%)		0.022		0.979	
1990–2003	136 (15.9)		56 (22.5)		56 (22.5)
2004–2011	294 (34.3)		89 (35.7)		91 (36.5)
2012–2019	428 (49.9)		104 (41.8)		102 (41.0)
Income, n (%)		0.551		0.551	
< $50,000	102 (11.9)		29 (11.6)		77 (30.9)
$50,000–64,999	267 (31.1)		69 (27.7)		33 (13.3)
≥ $65,000	489 (57.0)		151 (60.6)		139 (55.8)
Area, n (%)		0.860		0.887	
Metropolitan counties	762 (88.8)		222 (89.2)		220 (88.4)
Nonmetropolitan counties	95 (11.1)		27 (10.8)		29 (11.6)
UNK	1 (0.1)		0 (0.0)		0 (0.0)
Tumor size (mm), mean ± SD	71.17 ± 45.47	< 0.001	95.40 ± 58.45	0.469	91.62 (58.16)
T stage, n (%)		< 0.001		0.754	
T1	57 (6.6)		8 (3.2)		11 (4.4)
T2	282 (32.9)		52 (20.9)		59 (23.7)
T3	356 (41.5)		97 (39.0)		91 (36.5)
T4	163 (19.0)		92 (36.9)		88 (35.3)
N stage, n (%)		0.132		0.726	
N0	816 (95.1)		230 (92.4)		233 (93.6)
N1	42 (4.9)		19 (7.6)		16 (6.4)
M stage, n (%)		< 0.001		1.000	
M0	758 (88.3)		190 (76.3)		191 (76.7)
M1	100 (11.7)		59 (23.7)		58 (23.3)
Grade, n (%)		0.060		0.976	
I	143 (16.7)		44 (17.7)		43 (17.3)
II	136 (15.9)		32 (12.9)		30 (12.0)
III	31 (3.6)		13 (5.2)		15 (6.0)
IV	45 (5.2)		24 (9.6)		21 (8.4)
UNK	503 (58.6)		136 (54.6)		140 (56.2)
Mitotic rate, n (%)		0.088		0.747	
Low	384 (44.8)		92 (36.9)		91 (36.5)
High	110 (12.8)		35 (14.1)		41 (16.5)
UNK	364 (42.4)		122 (49.0)		117 (47.0)
Tumor site, n (%)		0.001		0.784	
Jejunum	604 (70.4)		148 (59.4)		152 (61.0)
Ileum	254 (29.6)		101 (40.6)		97 (39.0)
Chemotherapy, n (%)		0.020		0.322	
Yes	334 (38.9)		118 (47.4)		106 (42.6)
No/UNK	524 (61.1)		131 (52.6)		143 (57.4)
Lymphadenectomy, n (%)		0.029		0.716	
No/UNK^a^	560 (65.3)		143 (57.4)		148 (59.4)
Yes	298 (34.7)		106 (42.6)		101 (40.6)

*PSM* propensity score matching, *RR* radical resection, *LR* local resection, *SD* standard deviation *UNK* unknown

^a^Unknown lymphadenectomy status is limited to five (0.6%) patients of the LR group before PSM

Before PSM, LR and RR groups have significant differences in several variables (Table 1). The LR group had significantly smaller tumor and less T4 cases than the RR group. Patients in the LR group had less distant metastasis (M1) and ileal tumor, while they less frequently underwent chemotherapy and lymphadenectomy. In addition, marital status and year of diagnosis also differed significantly between groups. Therefore, we matched 249 patients in the LR group with 249 patients in the RR group based on propensity score.

After PSM, distributions of propensity score were similar between the two groups, and absolute standardized mean differences of all variables were < 0.1 (Supplementary Fig. 2). Comparison after PSM also showed the two groups not different significantly in all variables (*p* > 0.2), confirming a satisfactory balance (Table 1).

### Survival analysis

**Fig. 2:**
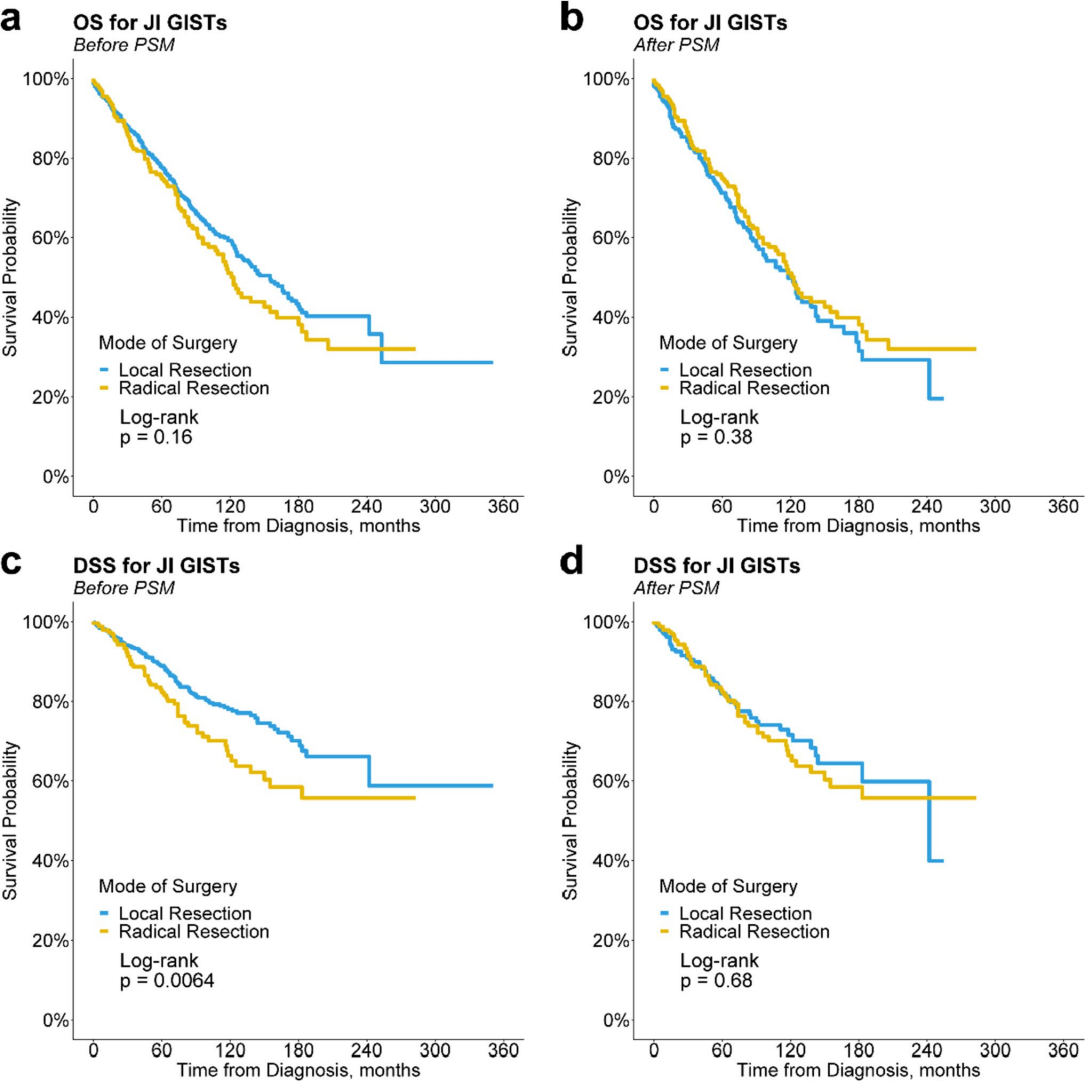
Kaplan–Meier curves of overall-survival (OS) and disease-specific survival (DSS) for patients with jejunoileal gastrointestinal stromal tumors (JI GISTs) in the local resection and radical resection groups. **a** OS before propensity score matching (PSM); **b** OS after PSM; **c** DSS before PSM; **d** DSS after PSM

Before PSM, LR group had similar OS (*p* = 0.16, Fig. 2a) but significantly better DSS (*p* = 0.0064, Fig. 2c) compared with the RR group. However, there were no significant differences between the LR and RR groups in long-term OS (5-year: 71.2% vs. 74.7%, *p* = 0.427; 10-year: 49.8% vs. 51.0%, *p* = 0.828) and DSS (5-year: 81.9% vs. 82.2%, *p* = 0.951; 10-year: 71.5% vs. 66.3%, *p* = 0.357) after PSM. KM analysis also showed that OS (HR 1.13, 95% CI 0.86–1.48, *p* = 0.382) and DSS (HR 0.92, 95% CI 0.64–1.34, *p* = 0.681) of the LR group was similar to that of the RR group (Fig. 2b, d).

### Subgroup sensitivity analyses

**Fig. 3:**
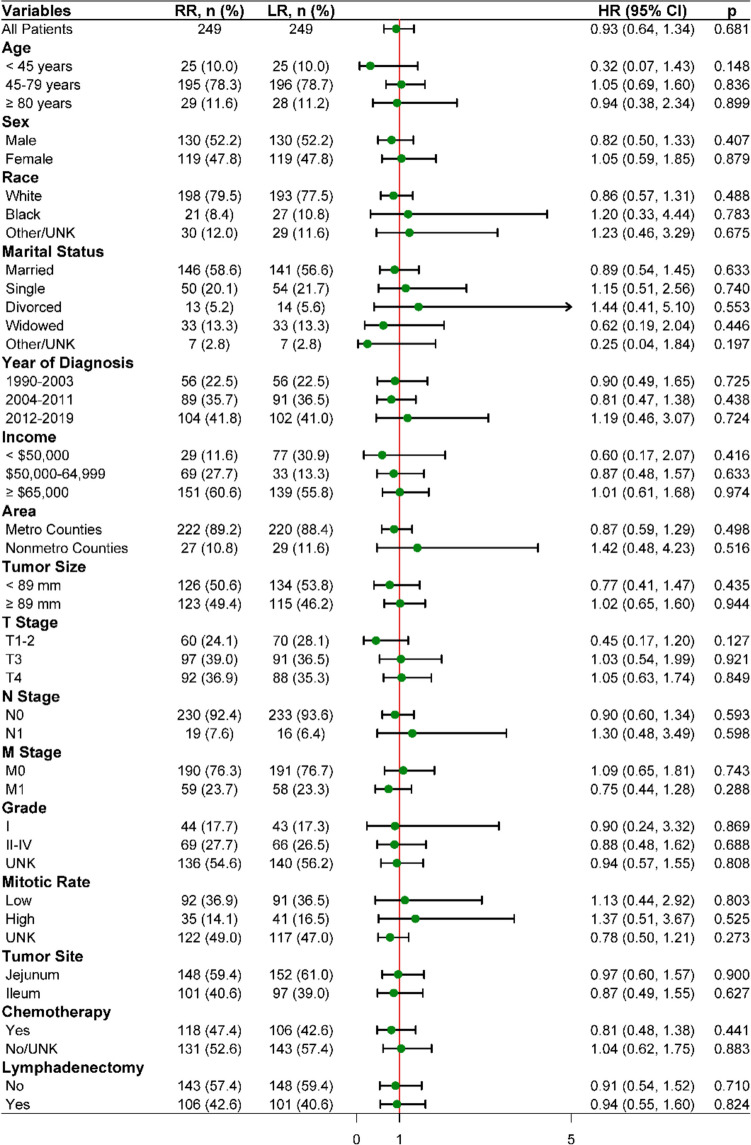
Hazard ratios (HRs) for disease-specific survival (DSS) with 95% confidence intervals (CIs) of radical resection (RR) and local resection (LR) groups in all patients and the subgroups, assumed by log-rank test. An HR < 1 implies a better DSS of LR group than RR group whereas > 1 implies the opposite. UNK: unknown; Metro: metropolitan; Nonmetro: nonmetropolitan

To further analyze the effects on DSS of LR and RR concomitant with other factors, subgroup log-rank tests were performed. The results showed that the LR group had similar DSS compared with the RR group across all subgroups (*p* > 0.05), which was consistent with the results of the overall matched population (Fig. 3).

### Risk factors for disease-specific survival

Cox proportional hazards regression model was then used to identify the risk factors for DSS of JI GISTs. Univariate Cox analysis revealed that age, marital status, year of diagnosis, income, tumor size, T stage, N stage, M stage, grade, mitotic rate and chemotherapy were significantly associated with DSS for patients with JI GISTs (Table 2). In addition, lymphadenectomy also tended to increase the risk of DSS (*p* = 0.058).

**Table 2:** Univariate and multivariate Cox proportional hazards analyses of the risk factors for survival in patients with JI GISTs

**Variable**	**Univariate analysis**		**Multivariate analysis**	
	**HR (95% CI)**	***p* value**	**HR (95% CI)**	***p* value**
**Age**				
< 45 years	Reference		Reference	
45–79 years	2.23 (1.03, 4.83)	0.043	2.12 (0.97, 4.66)	0.060
≥ 80 years	4.73 (1.97, 11.36)	0.001	5.70 (2.32, 13.99)	< 0.001
**Sex**				
Male	Reference			
Female	0.78 (0.54, 1.14)	0.203		
**Race**				
White	Reference			
Black	0.81 (0.41, 1.62)	0.555		
Other/UNK	1.22 (0.71, 2.07)	0.473		
**Marital status**				
Married	Reference			
Single	1.11 (0.70, 1.78)	0.656		
Divorced	2.49 (1.28, 4.87)	0.007		
Widowed	0.92 (0.48, 1.75)	0.801		
Other/UNK	2.17 (0.79, 5.96)	0.134		
**Year of diagnosis**				
1990–2003	Reference			
2004–2011	0.80 (0.53, 1.21)	0.295		
2012–2019	0.49 (0.27, 0.88)	0.018		
**Income**				
< $50,000	Reference			
$50,000–64,999	0.50 (0.25, 1.00)	0.051		
≥ $65,000	0.63 (0.43, 0.94)	0.022		
**Area**				
Metropolitan counties	Reference			
Nonmetropolitan counties	0.96 (0.54, 1.72)	0.903		
**Tumor size**				
< 89 mm	Reference		Reference	
≥ 89 mm	2.53 (1.71, 3.76)	< 0.001	1.74 (1.16, 2.62)	0.008
**T stage**				
T1-2	Reference			
T3	1.42 (0.79, 2.56)	0.242		
T4	2.99 (1.72, 5.20)	< 0.001		
**N stage**				
N0	Reference			
N1	2.49 (1.46, 4.23)	0.001		
**M stage**				
M0	Reference		Reference	
M1	4.27 (2.93, 6.21)	< 0.001	4.55 (3.07, 6.75)	< 0.001
**Grade**				
I	Reference			
II-IV	3.00 (1.46, 6.17)	0.003		
UNK	2.09 (1.04, 4.22)	0.040		
**Mitotic rate**				
Low	Reference		Reference	
High	2.49 (1.26, 4.93)	0.009	2.00 (1.00, 3.98)	0.049
UNK	2.14 (1.25, 3.67)	0.006	1.98 (1.15, 3.43)	0.014
**Tumor site**				
Jejunum	Reference			
Ileum	1.07 (0.74, 1.57)	0.709		
**Chemotherapy**				
Yes	Reference			
No/UNK	0.64 (0.44, 0.93)	0.020		
**Lymphadenectomy**				
No	Reference		Reference	
Yes	1.43 (0.99, 2.08)	0.058	1.59 (1.09, 2.33)	0.016
**Mode of surgery**				
Radical resection	Reference		Reference	
Local resection	0.93 (0.64, 1.34)	0.682	0.97 (0.66, 1.41)	0.858

*HR* hazard ratio, *CI* confidence interval, *UNK* unknown

All possible combinations of the variables above, together with mode of surgery, were then included in the multivariate analyses to study their interaction and relative contributions. Through model selection, a set of 17 models with ΔAICc < 2 was created. Three models had the least number of variables, among which Model 8 was considered the best with the relatively lowest AICc (AICc = 1149.8, ΔAICc = 1.06) which included age, lymphadenectomy, M stage, tumor size and mitotic rate (Supplementary Table 2). These five variables were further included in the multivariate Cox analysis together with mode of surgery, to prove their effects on DSS.

**Fig. 4:**
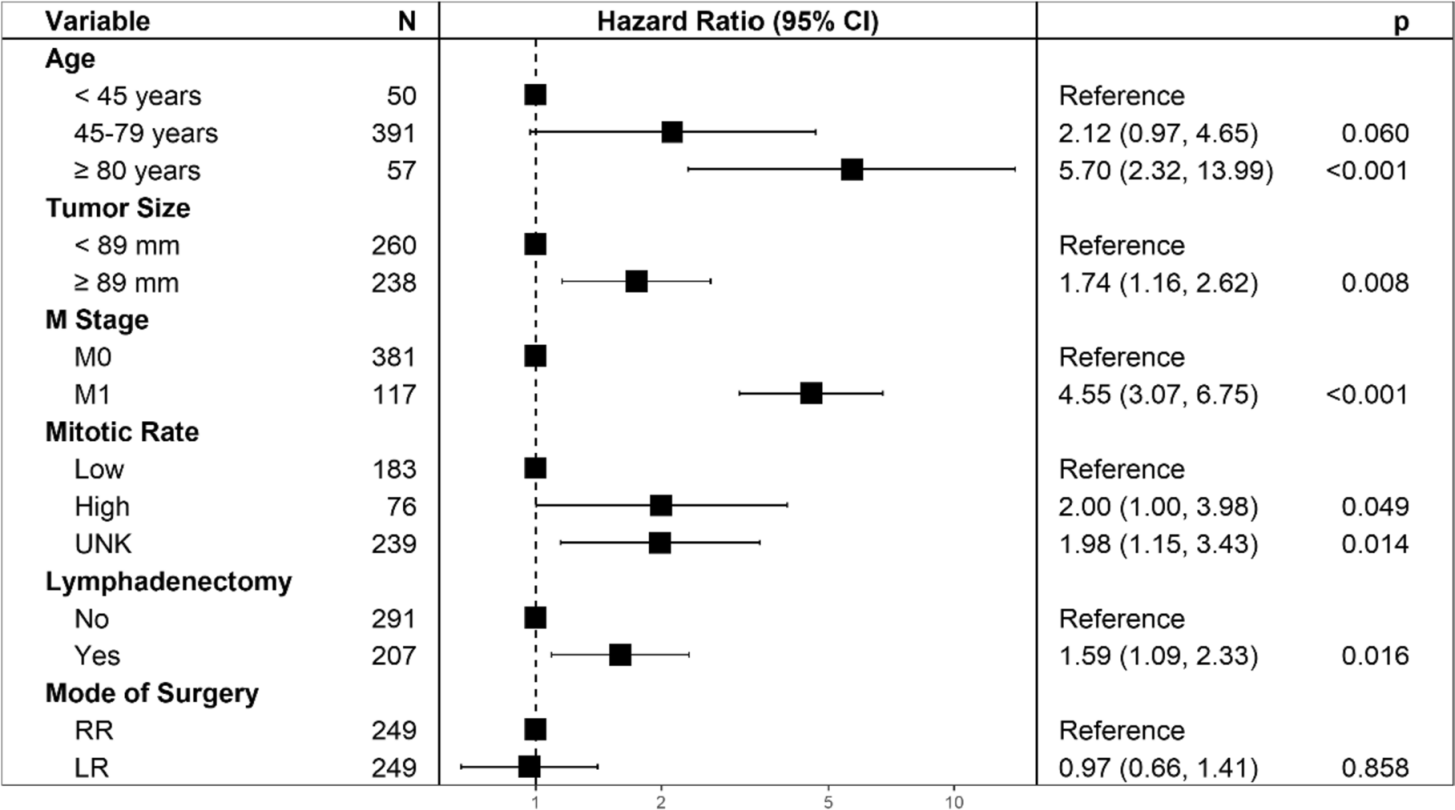
Hazard ratios with 95% confidence intervals (CIs) estimated by multivariate Cox regression analysis of prognostic factors for disease-specific survival in patients with jejunoileal gastrointestinal stromal tumors

In multivariate Cox analysis, there was no significant difference between RR and LR on DSS (HR 0.97, 95% CI 0.66–1.41, *p* = 0.858, Table 2, Fig. 4). Significant increased risk of DSS was associated with older age (≥ 80 years), larger tumor size (≥ 89 mm), distant metastasis (M1), high and unknown mitotic rate, and lymphadenectomy (Table 2, Fig. 4).

## Discussion

In this SEER-based matched cohort study, we found that patients with JI GISTs receiving LR has comparable survival outcome (both OS and DSS) to those receiving RR. Lymphadenectomy, age, tumor size, distant metastasis, mitotic rate, but not LR, were identified as independent prognostic factors for JI GISTs. Comparable survival outcome was accordant with these risk factors or in subgroup sensitivity analyses.

Although LR is recommended as first choice nowadays, patients with GISTs mainly underwent RR before the era of imatinib [1, 19]. Coincidently, we observed an increasing trend of LR in JI GISTs during the last three decades (1990–2003: 70.8%; 2004–2011: 76.8%; 2012–2019: 80.5%). Previous studies have shown that LR including endoscopic resection has advantages of function preserving, less complications, faster postoperative recovery and noninferior long-term survival compared with RR for GISTs [15, 17, 20]. Similarly, these findings have also been verified in population-based studies [16, 21–23]. However, the effect of LR on survival remains unknown for JI GISTs so far.

For JI tumors, RR with extensive bowel resection may lead to short bowel syndrome, a malabsorptive disorder with severe fatigue and gastrointestinal symptoms [24]. In addition, RR in distal ileum often involves resection of the ileocecal valve, leading to postoperative chronic diarrhea [25]. Preservation of mesenteric vessels has also been shown to reduce postoperative complications in JI tumors [26]. However, although RR is related with more postoperative complications and lower quality of life (QOL), it is performed instead of LR in many JI tumors from an oncologic point of view [27]. On the contrary, our study revealed that LR has comparable effect on both OS and DSS to RR for patients with JI GISTs (Fig. 2). Consistently, this result was observed within all subgroups (Fig. 3), as well as in multivariate analysis (Fig. 4). Considering that LR reduces complications and improves QOL, our findings implied that surgeons can choose LR in most JI GISTs patients without concerns for oncologic outcomes.

Interestingly, patients receiving LR has significant better DSS than those receiving RR before PSM (*p* = 0.0064, Fig. 2c). However, this finding should be attributed to the selection bias of surgical modality. From the unbalanced variables before PSM (Table 1), we could infer that surgeons tend to perform LR in patients with less aggressive tumors (having smaller size and more locoregional stage). In addition, we observed that LR was performed more frequently for ileal GISTs, probably due to the anatomical complexity of jejunum, especially the proximal part [28].

Tumor size, distant metastasis and mitotic rate have been proved to be prognostic factors for GISTs and included in several risk stratification systems [29]. Our study found the similar result that JI GISTs patients with tumor size ≥ 89 mm, distant metastasis, or high/unknown mitotic rate had worse survival. In addition, we also identified age and lymphadenectomy as independent prognostic factors for JI GISTs.

The prognostic role of age remains controversial for GISTs. Researchers found that older age is significantly correlated with poorer prognosis in retrospective studies [30–32], while others found it insignificant [33]. In multivariate analysis, compared with young patients (< 45 years old), patients over 80 years old had significant worse DSS (*p* < 0.001) but middle-age (45–79 years) also tended to be associated with poor prognosis (*p* = 0.060). In fact, recent studies suggested that GISTs might have distinct biology in younger patients [34]. The predictor effects of age on survival and the biological features of GISTs in different age groups are worth study.

Though discouraged for prophylactical purpose by most guidelines, performing lymphadenectomy in GISTs is controversial in case of enlarged regional lymph nodes [4, 35]. Previous SEER-based study found that lymphadenectomy is associated with poor survival of GISTs but the association was not observed in small intestinal GISTs [36]. We reported that lymphadenectomy is also a prognostic risk factor for JI GISTs (*p* = 0.016) and might be considered harmful. Nevertheless, this result needs further validation in at a finer scale studies given the limitation of population-based study.

There are some limitations in this SEER-based study. Firstly, the main limitation is that SEER database has shortcomings in information depth, lacking several important factors such as intraabdominal tumor rupture, margin status, and genetic mutation of KIT or PDGFRA. Although these factors are known to be associated with prognosis [1, 35, 37], inability to study them might influence our analysis. Furthermore, although integration of surgery with adjuvant imatinib therapy for GISTs has been proved to improve survival significantly, lack of information on regimen of chemotherapy in SEER Database hampered further investigation of imatinib in our cohort, underestimating its effect on survival. Secondly, this study did not include small intestinal GISTs with overlapping lesions, located at unspecified site, or located at Meckel diverticulum. However, given the rare incidence of these tumor, our findings might be generalized to most types of JI GISTs. Thirdly, GISTs might not be accurately diagnosed before GISTs-specific histologic code was applied in 2001. In early 2000s, the identification of GIST was fortified due to the progress in immunohistochemical diagnosis [38, 39]. However, 93.9% of our patients were diagnosed after 2001, ensuring a cohort with mostly reliable diagnosis. Lastly, our analysis had some biases inevitably as a retrospective study. However, while previous SEER-based studies used multivariate regression or PSM [21–23], the combination of both methodologies in our analysis further minimized the biases. Further prospective studies, especially multi-center randomized controlled trials, are needed to verify the effect of LR on JI GISTs. Evidence found in our report can be applied to guide future study design.

In conclusion, our study found that LR has comparable survival outcome to RR in patients with JI GISTs. To the best of our knowledge, this is the first study that compares the effect of different surgical modes on survival for patients with JI GISTs. Compared with single-center studies, our results might be better extrapolated to the general population given a larger sample size and longer follow-up. LR can be carried out safely without compromising oncological outcome, and should be considered as a treatment option in selected patients with JI GISTs.

### Supplementary Information

Below is the link to the electronic supplementary material.

Supplementary file1 (TIFF 3725 KB)

Supplementary file2 (TIFF 4601 KB)

Supplementary file3 (DOCX 29 KB)

## Data Availability

The datasets in this study were generated using data obtained from the SEER database, and are available at https://doi.org/10.6084/m9.figshare.22360540.v1.
