# Supplementary figures and images for: Survival outcome of local versus radical resection for jejunoileal gastrointestinal stromal tumors: a propensity score-matched population-based analysis
Source: Int J Colorectal Dis. 2023 Oct 19;38(1):253. doi: 10.1007/s00384-023-04548-w (PMC10587263; doi:10.1007/s00384-023-04548-w)

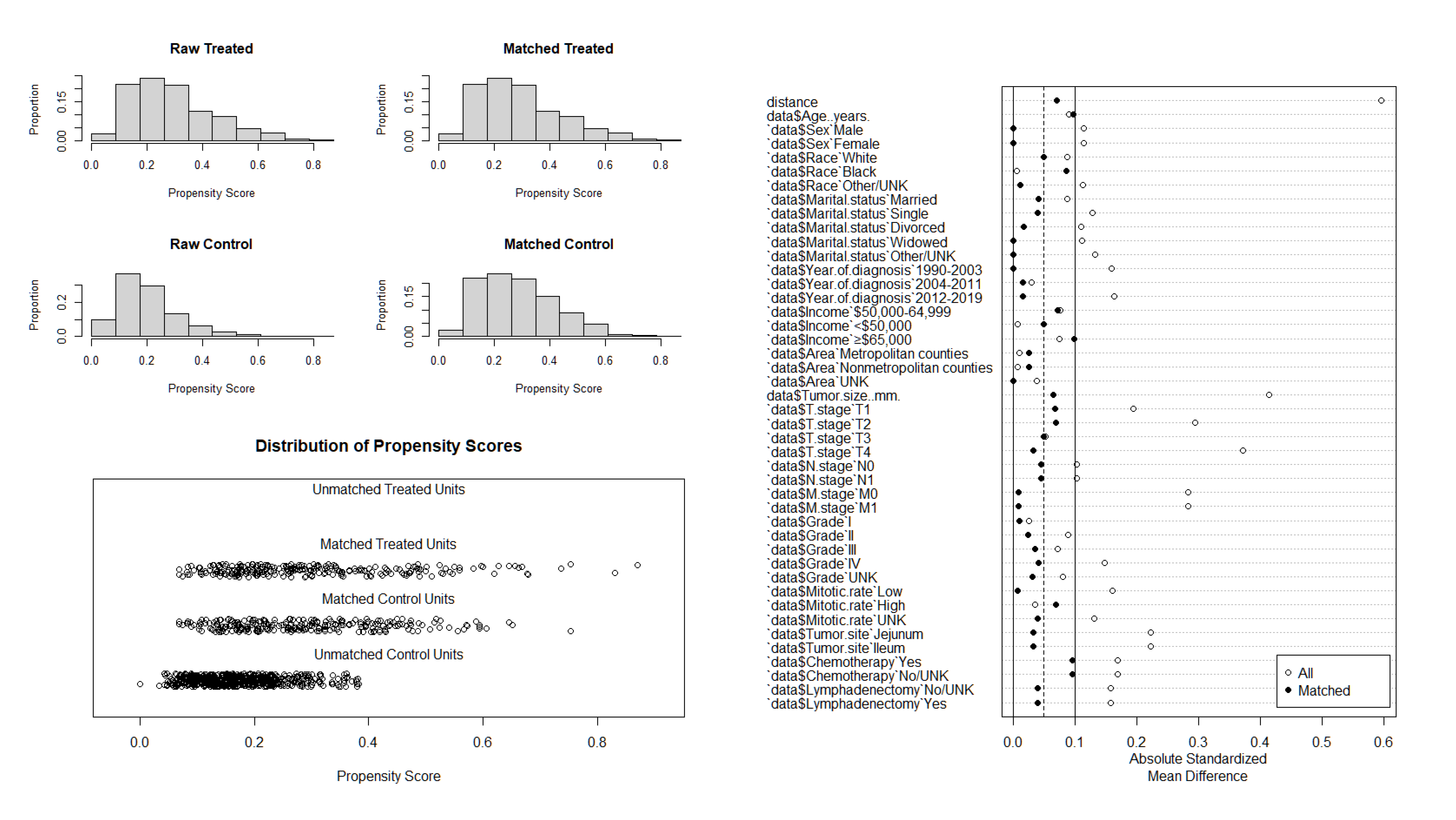

Supplement: Supplementary file 2 — Supplementary file2 (TIFF 4601 KB) [file 384_2023_4548_MOESM2_ESM.tiff]
