# Supplementary material for: Survival outcome of local versus radical resection for jejunoileal gastrointestinal stromal tumors: a propensity score-matched population-based analysis
Source: Int J Colorectal Dis. 2023 Oct 19;38(1):253. doi: 10.1007/s00384-023-04548-w (PMC10587263; doi:10.1007/s00384-023-04548-w)
Supplement: Supplementary file 3 — Supplementary file3 (DOCX 29 KB) [file 384_2023_4548_MOESM3_ESM.docx]

**Supplementary Table 1** Patient selection in SEER database using SEER*Stat software version 8.4.0.1

|  | **Database Name** | **Selection Statement** |
| --- | --- | --- |
| SEER-8 | Incidence - SEER Research Plus Data, 8 Registries, Nov 2021 Sub (1975-2019) | {Site and Morphology.Primary Site - labeled} = 'C17.1-Jejunum','C17.2-Ileum'  AND {Site and Morphology.ICD-O-3 Hist/behav} = '8936/3: Gastrointestinal stromal sarcoma'  AND {Age at Diagnosis.Age recode with <1 year olds} != 'Unknown'  AND {Site and Morphology.Behavior code ICD-O-3} = 'Malignant'  AND {Race, Sex, Year Dx, Registry, County.Year of diagnosis} = '1975','1976','1977','1978','1979','1980','1981','1982','1983','1984','1985','1986','1987','1988','1989','1990','1991' |
| SEER-12 | Incidence - SEER Research Plus Data, 12 Registries, Nov 2021 Sub (1992-2019) | {Site and Morphology.Primary Site - labeled} = 'C17.1-Jejunum','C17.2-Ileum'  AND {Site and Morphology.ICD-O-3 Hist/behav} = '8936/3: Gastrointestinal stromal sarcoma'  AND {Age at Diagnosis.Age recode with <1 year olds} != 'Unknown'  AND {Site and Morphology.Behavior code ICD-O-3} = 'Malignant'  AND {Race, Sex, Year Dx, Registry, County.Year of diagnosis} = '1992','1993','1994','1995','1996','1997','1998','1999' |
| SEER-17 | Incidence - SEER Research Plus Data, 17 Registries, Nov 2021 Sub (2000-2019) | {Site and Morphology.Primary Site - labeled} = 'C17.1-Jejunum','C17.2-Ileum'  AND {Site and Morphology.ICD-O-3 Hist/behav} = '8936/3: Gastrointestinal stromal sarcoma'  AND {Age at Diagnosis.Age recode with <1 year olds} != 'Unknown'  AND {Site and Morphology.Behavior code ICD-O-3} = 'Malignant'  AND {Race, Sex, Year Dx.Year of diagnosis} = '2000','2001','2002','2003','2004','2005','2006','2007','2008','2009','2010','2011','2012','2013','2014','2015','2016','2017','2018','2019' |

**Supplementary Table 2** Prognostic models for jejunoileal GISTs with ΔAICc < 2 created by automated model selection.

| **Model** | **Age** | **Lnd** | **M** | **Siz** | **Mrs** | **Yrd** | **Grd** | **Mtr** | **Inc** | **Cht** | **N** | **T** | **Sur** | **df** | **logLik** | **AICc** | **ΔAICc** | **AICcWt** |
| --- | --- | --- | --- | --- | --- | --- | --- | --- | --- | --- | --- | --- | --- | --- | --- | --- | --- | --- |
| 1 | + | + | + | + | + | + | + |  |  |  |  |  |  | 13 | -559.500 | 1148.7 | 0 | 0.024 |
| 2 | + | + | + | + | + | + |  |  | + |  |  |  |  | 13 | -559.711 | 1149.1 | 0.42 | 0.020 |
| 3 | + | + | + | + | + |  |  | + |  |  |  |  |  | 11 | -562.341 | 1149.3 | 0.61 | 0.018 |
| 4 | + | + | + | + | + | + |  |  |  |  |  |  |  | 11 | -562.431 | 1149.5 | 0.79 | 0.016 |
| 5 | + | + | + | + | + |  | + | + |  |  |  |  |  | 13 | -559.944 | 1149.6 | 0.89 | 0.016 |
| 6 | + | + | + | + |  | + | + |  |  |  |  |  |  | 9 | -564.960 | 1149.7 | 0.97 | 0.015 |
| 7 | + | + | + | + |  |  | + | + |  |  |  |  |  | 9 | -564.985 | 1149.7 | 1.02 | 0.015 |
| 8 | + | + | + | + |  |  |  | + |  |  |  |  |  | 7 | -567.347 | 1149.8 | 1.06 | 0.014 |
| 9 | + | + | + | + | + | + | + |  |  | + |  |  |  | 14 | -558.733 | 1149.8 | 1.08 | 0.014 |
| 10 | + | + | + | + | + | + | + |  | + |  |  |  |  | 15 | -557.421 | 1149.8 | 1.13 | 0.014 |
| 11 | + | + | + | + | + |  |  | + | + |  |  |  |  | 13 | -560.145 | 1150.0 | 1.29 | 0.013 |
| 12 | + | + | + | + | + |  | + |  |  |  |  |  |  | 11 | -562.756 | 1150.2 | 1.44 | 0.012 |
| 13 | + | + | + | + | + | + |  |  | + | + |  |  |  | 14 | -558.950 | 1150.2 | 1.52 | 0.011 |
| 14 | + | + | + | + |  | + |  |  |  |  |  |  |  | 7 | -567.611 | 1150.3 | 1.58 | 0.011 |
| 15 | + | + | + | + |  |  | + |  |  |  |  |  |  | 7 | -567.621 | 1150.3 | 1.60 | 0.011 |
| 16 | + | + | + | + | + | + |  |  |  | + |  |  |  | 12 | -561.633 | 1150.4 | 1.70 | 0.010 |
| 17 | + | + | + | + | + |  |  | + |  | + |  |  |  | 12 | -561.735 | 1150.6 | 1.91 | 0.009 |

*GISTs* gastrointestinal stromal tumors, *AICc* corrected Akaike Information Criterion, *ΔAICc* difference in AICc between a model and the model with the lowest AICc, *Lnd* lymphadenectomy, *M* M stage, *Siz* tumor size, *Mrs* marital status, *Yrd* year of diagnosis, *Grd* grade, *Mtr* mitotic rate, *Inc* income, *Cht* chemotherapy, *N* N stage, *T* T stage, *Sur* surgery, *df* degrees of freedom, *logLik* log-Likelihood, *AICcWt* AICc weights represent values approximate the likelihood that a given model is the best of those in the set
